# RISKS FOR LATER-LIFE COGNITIVE IMPAIRMENT: DISENTANGLING CHILDHOOD AND EARLY FAMILY-LIFE PREDICTORS

**DOI:** 10.1093/geroni/igac059.3040

**Published:** 2022-12-20

**Authors:** Lindsey Mundy, Kimson Johnson, Jacqui Smith

**Affiliations:** University of Michigan, Tuscaloosa, Alabama, United States; University of Michigan, Ann Arbor, Michigan, United States; University of Michigan, Ann Arbor, Michigan, United States

## Abstract

Observing later-life health outcomes using a life-course model uncovers critical periods of influence. We investigated the relationship between five multi-component measures of childhood disadvantage (determined by Morton et al., 2022) and risk of cognitive impairment (determined by the Langa-Weir Classification) in later life. Data from the Health and Retirement Study (n= 9,509) were used. We conducted a multivariable parametric survival analysis to examine the relationships between the early life predictors of low socioeconomic status (SES), childhood impairments, risky adolescent behaviors, risky parental behaviors, and childhood chronic illness and the risk of developing later-life cognitive impairment between 2006 and 2016. We found that each additional indicator of low SES, risky adolescent behavior, and impairment in childhood increased the risk of developing cognitive impairment over the 10 years studied (HR=1.14, p < .05; HR=1.15, p < .05; HR=1.19, p < .05). Additional indicators of risky parental behaviors, however, were related to a smaller risk of developing cognitive impairment over time (HR=0.96, p < .05). Childhood chronic illness was not significantly associated with the risk of developing cognitive impairment. These results show that early-life factors might have long-term implications for the development of cognitive impairment in later life and display differential relationships with risk when parsed into competing categories. Measures of disadvantage indicating smaller risk may point to the resilience and plasticity of the brain in early life and highlight the need for the study of multiple life stages in order to track predictors of cognitive impairment throughout the life course.

